# Ancillary Findings on CT Pulmonary Angiograms that are Negative for Pulmonary Embolism

**DOI:** 10.51894/001c.11769

**Published:** 2020-01-30

**Authors:** Paul Stein, Fadi Matta, Brett J. Gerstner, Edward J. Kakish, Patrick G. Hughes, Julie Lata, Christopher C. Trigger, Kevin A. Jutzy, Michael Q. Doyle, Mathew A. Warpinski, William D. Corser, Jerome P. Long, Nasheed S. Fakhouri, Corion L. Jones, Kristen N. Owen, Casey Lyons, Damien Carracedo, Ian P. Skinner, Laura A. Warner, Ethan R. Saffer, Brody A. Deming, Keith D. Cronovich, Mary J. Hughes

**Affiliations:** 1 Osteopathic Medical Specialties, College of Osteopathic Medicine Michigan State University; 2 Osteopathic Medical Specialties Michigan State University; 3 Emergency Medicine Sparrow Health; 4 Emergency Medicine University of Toledo Medical Center; 5 Integrated Medical Science Florida Atlantic University; 6 Emergency Medicine McLaren Macomb Hospital; 7 Emergency Medicine Spectrum Lakeland Health; 8 Emergency Medicine Henry Ford Macomb; 9 Emergency Medicine McLaren Oakland; 10 Emergency Medicine Beaumont Hospital; 11 College of Osteopathic Medicine Statewide Campus System Michigan State University; 12 Emergency Medicine Spectrum Lakeland; 13 Emergency Medicine University Toledo Medical Center; 14 Emergency Medicine Sparrow Health System; 15 College of Medicine, Department of Emergency Medicine Florida Atlantic University; 16 Emergency Medicine Spectrum Health Lakeland Hospital

**Keywords:** pulmonary angiography, ct angiography, pulmonary embolism

## Abstract

**CONTEXT:**

One advantage of computed tomographic pulmonary angiograms (CTPA) is that they often show pathology in patients in whom pulmonary embolism (PE) has been excluded. In this investigation, we identified the ancillary findings on CTPAs that were negative for PE to obtain an impression of the type of findings shown.

**METHODS:**

This was a retrospective analysis of findings on CTPAs that were negative for PE obtained in nine emergency departments between January 2016 - February 2018. Ancillary findings were assessed by review of the radiographic reports.

**RESULTS:**

Ancillary findings were identified in N=338 (40.9%) of 825 patients with CTPAs that were negative for PE. Most ancillary findings, 254 (75.1%) of 338 were pulmonary or pleural abnormalities. Liver, gall bladder, kidney, or pancreatic abnormalities were shown in 26 (7.7%) cases, and abnormalities of the heart or great vessels were shown in 23 (6.8%) of cases. Abnormalities of the esophagus or intestine were shown in 12 (3.6%), abnormalities of the thyroid in 10 (3.0%) and abnormalities of bone or soft tissue lesions were shown in three (0.9%) cases. Inferential statistical procedures demonstrated that the occurrence of ancillary findings in patients with negative CTPAs was proportionately greater in patients who were 50 years and older (p < 0.001), although not between genders (p = 0.145).

**CONCLUSIONS:**

Ancillary findings on CTPAs that were negative for PE were frequently reported. Future studies might focus of the extent to which ancillary findings on CTPA assisted physicians in management of the patient.

## INTRODUCTION

Pulmonary embolism (PE) is obstruction of a pulmonary artery or one or more of its branches that is produced by a thrombus (blood clot). Generally, PE’s originate in a vein of the leg or pelvis and travel through the veins, right atrium and right ventricle to the pulmonary artery and/or its branches. Signs and symptoms are nonspecific^1^ and the diagnosis is made by appropriate imaging techniques, usually computed tomographic pulmonary angiography (CTPA). Computed tomographic pulmonary angiography is a minimally invasive radiographic procedure in which images of the pulmonary arteries are generated by synthesis of x-ray transmission data obtained in many different directions in a given plane. It is minimally invasive, in that only an intravenous injection of contrast material is required. The risks of conventional pulmonary angiography for the diagnosis of PE,^2^ which requires catheterization of the pulmonary arteries, are eliminated. Still, with CTPA there remain the risks of allergy and nephrotoxicity from contrast material^3^ and the risks associated with ionizing radiation.^4^

The first evaluation of contrast–enhanced CTPA compared with pulmonary angiography was published in 1992 and based on results with single detector units.^5^ In 2000, the use of CTPA began to increase and by 2001 the use of CTPA exceeded the use of ventilation/perfusion lung scans.^6^ Conventional pulmonary angiography is now rarely used, and in 2010, none of the reference tests for PE in the Prospective Investigation of Pulmonary Embolism Diagnosis III (PIOPED III) investigation of magnetic resonance angiography for PE used conventional pulmonary angiography as the reference test.^7^

Evaluation of multidetector CTPA for the diagnosis of PE was published in 2006 in the Prospective Investigation of Pulmonary Embolism Diagnosis II (PIOPED II).^8^ In 2006, 88% of hospitals in the United States with ≥ 25 beds had CT scanners and 39% had multidetector CT scanners.^9^ By 2014, multidetector CT scanners were available in virtually all hospitals in the United States.^10^ Although CTPA is the usual imaging test for PE, interpretation is subject to error, and PE may be overdiagnosed, particularly if the PE appears to be limited to a solitary segmental or subsegmental branch.^8^

Radiation exposure is an important consideration when using CTPA.^4^ A small but measurable increased risk of breast or lung cancer has been reported in patients in whom only one CTPA was obtained, especially in younger women.^4,11^ When considering radiation dose, whole body radiation with a chest posterior-anterior (PA) radiograph is 0.02 mSv^12^, whereas with 64-detector CTPA it is 19.1 mSv.^13^ Therefore, the whole body radiation from a single CTPA with a 64-detector unit would be equivalent to receiving 955 chest PA radiographs.

Best practice advice from the American College of Physicians for evaluation of patients with suspected acute PE has been to estimate the probability of PE based on clinical prediction rules in combination with a high–sensitivity D-dimer if the probability is intermediate or low but does not meet all of the Pulmonary Embolism Rule-Out Criteria.^14^

Sensitive D-dimer assays include the enzyme–linked immunosorbent assay (ELISA), quantitative rapid ELISA, semiquantitative rapid ELISA, qualitative rapid ELISA, and quantitative latex agglutination.^15^ The whole blood agglutination test has the lowest sensitivity, 78%.^15^ The quantitative latex agglutination (89% sensitive) and semiquantitative latex agglutination (92% sensitive) assays are less sensitive than the ELISA assays (93%-95% sensitive), but more sensitive than the whole blood agglutination assay.^15^

A PE diagnosis can be safely excluded by using clinical prediction rules with D-dimer.^16,17^ The clinical prediction rules include the Wells score,^16,17^ the Geneva score,^18^ and the Pulmonary Embolism Rule-out Criteria (PERC).^19^ Their use in some patients eliminates the need for CTPA and its associated ionizing radiation.^16^ However, there has been suboptimal implementation of diagnostic algorithms and overuse of CTPA in patients with suspected PE.^20^

In 2016, a review of 16 investigations during the past decade showed negativity rates of CTPA in the United States between 90% and 92%.^20^ An investigation in 2019 showed 99% of CTPAs were negative.^21^ There are several reasons for nonadherence to established guidelines. In most settings, CTPA is available 24 hours a day, is reliable, is faster and easier to perform than a clinical history or D-dimer test and provides a quicker diagnostic answer.^22^ In comparison, clinical evaluation can be subjective, time consuming and exposes the attending physician to the risk of a missed diagnosis.^22^

An additional advantage of CTPA is that it often shows pathology in patients in whom PE has been excluded.^23–29^ However, the use of CTPA as a screening test has not been endorsed by the US Preventive Health Task Force or the American College of Radiology due to potential risks and uncertain benefits.^30^ Even so, based on the high negativity rate of CTPA, many physicians believe that the benefit of obtaining a CTPA exceeds the risk, and that one of the advantages, in addition to a quick diagnostic answer, is the possibility of the test showing a useful ancillary finding.^29,31^

The aim of this study was the further assessment of ancillary findings on CTPA that are negative for PE, the results of which may contribute to determining whether the findings are worth the risk of exposure to radiation and contrast material.

## METHODS

This was a retrospective analysis of CTPA findings that were negative for PE. There were nine participating centers. Before data collection, the institutional review boards at each participating center had approved the investigation. The CTPAs were evaluated from January 2016 through February 2018. All CTPA data were obtained from 16, 64, 80, 128, or 320-detector units. The primary advantage of 256- and 320-slice CT is the increased craniocaudal coverage. In a comparison of prospectively gated 64- and 256-slice CT scanning, the 256-slice scan provided better and more stable image quality, at equivalent effective radiation dose.^32^ Findings on CTPA reports were entered on data collection sheets.  Attending physicians (BJG, EJK, PGH, JL, CCT, KAJ, MQD, KNO, MAW, MJH), upper level residents (JPL, NSF, CL, DC, IPS, LAW, ERS, KDC) and in two instances technicians (CLJ, BAD) abstracted all charts and completed data abstraction sheets. Research associates or other lesser trained individuals did not make diagnosis-related decisions. In the analytic sample, we reported only one ancillary finding per patient.

If more than one ancillary finding was documented on the CTPA, we reported only the one assessed as being the most important. These decisions were made by first author (PDS) based on a hierarchy of findings that two of the authors (PDS, FM) developed for this investigation. Findings that would not have been clinically recognized, except perhaps on the chest radiograph (e.g., lung nodules or masses) were considered the most important. Nodules outside the lung such as thyroid nodules were considered less important. Atelectasis or pneumonia was considered more important than chronic disease such as emphysema which was likely to be already known.

**Figure 1:**
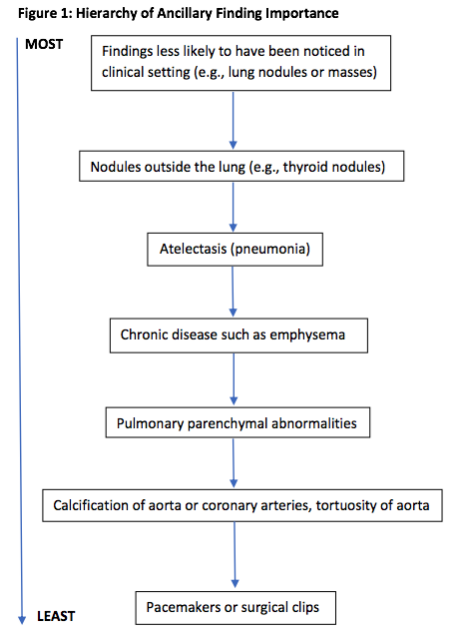
Hierarchy of Ancillary Finding Importance

Unless there was evidence of a malignancy, pulmonary findings were considered more important than bone findings. Pulmonary parenchymal abnormalities were considered more important than calcification of the aorta or coronary arteries or tortuosity of the aorta. Pacemakers or surgical clips were treated as the least important ancillary findings (Figure 1).

### Statistical analyses

Data were analyzed by author FM using SPSS Version 11.5 for Windows (SPSS Inc, Chicago, Illinois). Tests of equality of two proportions were carried out using the two-tailed Fisher exact test (http://www/graphpad.com/quickcalcs/contingency2.cfm). We considered P values of .05 or lower as significant. Continuous variables were reported as mean ± standard deviation.

## RESULTS

Data from CTPAs were obtained in N = 893 patients with suspected acute PE. The CTPAs were negative for PE in 825 (92.4%). A plain chest radiograph in those with negative CTPAs was obtained within 24 hours prior to the CTPA in 415 of 825 (50.3%) patients and not obtained in 410 of 825 (49.7%) patients. The mean age of the 825 patients with CTPA that were negative for PE was 56.0 years ± 18 years. The majority of patients were females, 536 (65.0%) (P < 0.0001), females versus males.

**Table 1.** Ancillary Findings on CT Pulmonary Angiograms of Patients with No Pulmonary Embolism

**Category**	**CT Angiographic Finding**	**Number**
Pulmonary or pleural		254 (75.1)
	Lung nodule, mass or density	63 (18.6)
	Atelectasis	46 (13.6)
	Emphysema	32 (9.5)
	Infiltrate	23 (6.8)
	Ground glass attenuation	27 (8.0)
	Pneumonia	9 (2.7)
	Consolidation	9 (2.7)
	Chronic interstitial disease	7 (2.1)
	Opacity	8 (2.4)
	Air bronchogram	3 (0.9)
	Granuloma	5 (1.5)
	Inflammation	1 (0.3)
	Congestion	2 (0.6)
	Low lung volume	1 (0.3)
	Bronchitis or peribronchial thickening	3 (0.9)
	Pleural effusion	15 (4.4)
Heart and great vessels		23 (6.8)
	Cardiomegaly	12 (3.6)
	Aortic aneurysm or ectatic aorta	4 (1.2)
	Dilated superior vena cava	2 (0.6)
	Dilated pulmonary trunk	1 (0.3)
	Pericardial effusion	3 (0.9)
Mediastinum		1 (0.3)
	Pneumomediastinum	1 (0.3)
Lymph node		9 (2.7)
	Lymph node, paratracheal or mediastinal	9 (2.7)
Liver, gall bladder, kidney, pancreas		26 (7.7)
	Fatty liver	12 (3.6)
	Liver cyst	1 (0.3)
	Cholelithiasis	4 (1.2)
	Cholecystitis	1 (0.3)
	Kidney cyst	4 (1.2)
	Renal calculi	1 (0.3)
	Kidney angiomyolipoma or adenoma	2 (0.6)
	Pancreas calcification	1 (0.3)
Thyroid		10 (3.0)
	Thyroid mass, nodule, thickening, or hypodensity	10 (3.0)
Esophagus, intestine		12 (3.6)
	Esophagitis	2 (0.6)
	Hiatal hernia	9 (2.7)
	Diverticular disease	1 (0.3)
Bone, soft tissue		3 (0.9)
	Bone lesion	2 (0.6)
	Soft tissue disease	1 (0.3)
Total		338 (100)

CT = computed tomographic

Among patients who had CTPAs that were negative for PE, ancillary findings were reported in 338 of 825 (40.9%). The majority, 254 of 338 (75.1%) of such ancillary findings were pulmonary or pleural abnormalities (Table 1). Many of the ancillary findings were unrelated to any clinical findings that could suggest PE. Liver, gall bladder, kidney, or pancreatic abnormalities were 26 (7.7%), abnormalities of the heart or great vessels 23 (6.8%), esophagus or intestine in 12 (3.6%), thyroid 10 (3.0%) and bone or soft tissue lesions three (0.9%) of 338 patients.

To compare the statistical significance of differences in ancillary findings among age and gender sample subgroups, one of the authors (WDC) first conservatively categorized sample patients’ ages into equivalent-sized tertile subgroups. Next, this analyst conducted a series of Chi Square and multivariate binary logistic regression statistical procedures to examine whether the occurrence of ancillary findings in negative CTPA patients varied significantly when stratifying them by age group and gender.

In summary, the occurrence of ancillary findings was significantly higher in the middle and older “50 through 65” (67.2%) and “66 through 97 years” (66.0%) subgroups when compared to the younger “18 through 49 years old” (47.4%) sample subgroup. (Pearson Chi-Square = 19.773, df 2, p < 0.001). However, proportionate ancillary finding differences between males and females were not found to be statistically significant. (Pearson Chi-Square = 2.214, df 1, p = 0.145). Similar results were obtained from the binary logistic regression model, with age group a statistically significant predictor of a {0,1} ancillary finding (Wald = 13.121, df 1, p <0.001) when controlling for the non-significant Gender model term (Wald = 1.174, df 1 p = 0.279).

## DISCUSSION

Among sample patients who underwent CTPAs for suspected PE, 825 (92.4%) cases were negative. Ancillary findings on the negative CTPAs were reported in 338 (40.9%). The prevalence of negative CTPAs that we observed was comparable with results of prior research groups that reported that 90-92% of CTPAs were negative for suspected PE.^20^ This indicates that physicians in emergency departments have a low threshold for obtaining CTPAs and may be overutilizing them, thereby unnecessarily exposing patients to ionizing radiation.

Although firm and documented conclusions on the carcinogenic potential of CTPAs are lacking, there is general agreement that the amount of radiation delivered to the mammary glands of women of reproductive age in the course of CTPA could substantially increase the incidence of breast cancer.^4,33^ The use of pretest clinical evaluation of symptoms would decrease the negativity rate of CTPAs.^16,17,21^ In the early 1990’s, prospective diagnostic trials that used pretest clinical evaluation of symptoms followed by lung scans showed PE in at least one-third of the patients examined.^34,35^

Time constraints imposed on emergency departments due to an increased focus on turnaround time and increased patient load may contribute to the failure of many emergency department physicians to follow the clinical guidelines related to risk stratification of patients.^21^ However, we and others showed frequent pathology on CTPAs in patients in whom PE was excluded.^23–29^ Whether the identified pathology outweighs the risk of radiation, particularly in women and younger patients,^4^ remains understudied.

The reason that more women than men in this sample had negative CTPAs is not clear. The average population–based incidence of PE in emergency departments from 2007-2012 was 120/100,000 women/year and 110/100,000 men/year (rate ratio 1.09).^36^ Rates of use of ventilation-perfusion lung scans and venous ultrasonography of the lower extremities were also higher in women.^37^ Perhaps in view of the higher prevalence of PE in women than men, physicians are more inclined to perform diagnostic tests. Perhaps women are more responsible healthcare consumers than men and present themselves earlier than men who may tend to “hold out longer.”

Whether the size of emergency department facilities (i.e., metro/urban/rural) or whether academic-based or community-based may influence results is another area for future research.

## CONCLUSIONS

In conclusion, most patients with suspected PE 825 (92.4%) had CTPAs that were negative for PE. A plain chest radiograph in those with negative CTPAs was obtained within 24 hours prior to the CTPA in only half 415/825 (50.3%). Ancillary findings on CTPAs that were negative for PE were reported in 338/825 (40.9%) of patients. The majority of ancillary findings 254/338 (75.1%) were pulmonary or pleural abnormalities. Many of the ancillary findings were unrelated to clinical findings that could suggest PE. Liver, gall bladder, kidney, or pancreatic abnormalities were shown in 26 (7.7%) of patients with ancillary findings, abnormalities of the heart or great vessels in 23 (6.8%), esophagus or intestine in 12 (3.6%), thyroid in 10 (3.0%), and bone or soft tissue lesions in 3 (0.9%) of patients with ancillary findings. The next step in assessing the issues raised from these results is to further examine the extent to which ancillary findings on CTPA assist emergency department physicians in managing patients with suspected PE.

### Conflict of Interest

The authors declare no conflict of interest.
